# An emerging role for misfolded wild-type SOD1 in sporadic ALS pathogenesis

**DOI:** 10.3389/fncel.2013.00253

**Published:** 2013-12-16

**Authors:** Melissa S. Rotunno, Daryl A. Bosco

**Affiliations:** Department of Neurology, University of Massachusetts Medical CenterWorcester, MA, USA

**Keywords:** amyotrophic lateral sclerosis (ALS), sporadic amyotrophic lateral sclerosis, SOD1, protein misfolding, immunotherapy

## Abstract

Amyotrophic lateral sclerosis (ALS) is a fatal neurodegenerative disorder that targets motor neurons, leading to paralysis and death within a few years of disease onset. While several genes have been linked to the inheritable, or familial, form of ALS, much less is known about the cause(s) of sporadic ALS, which accounts for ~90% of ALS cases. Due to the clinical similarities between familial and sporadic ALS, it is plausible that both forms of the disease converge on a common pathway and, therefore, involve common factors. Recent evidence suggests the Cu,Zn-superoxide dismutase (SOD1) protein to be one such factor that is common to both sporadic and familial ALS. In 1993, mutations were uncovered in SOD1 that represent the first known genetic cause of familial ALS. While the exact mechanism of mutant-SOD1 toxicity is still not known today, most evidence points to a gain of toxic function that stems, at least in part, from the propensity of this protein to misfold. In the wild-type SOD1 protein, non-genetic perturbations such as metal depletion, disruption of the quaternary structure, and oxidation, can also induce SOD1 to misfold. In fact, these aforementioned post-translational modifications cause wild-type SOD1 to adopt a “toxic conformation” that is similar to familial ALS-linked SOD1 variants. These observations, together with the detection of misfolded wild-type SOD1 within human post-mortem sporadic ALS samples, have been used to support the controversial hypothesis that misfolded forms of wild-type SOD1 contribute to sporadic ALS pathogenesis. In this review, we present data from the literature that both support and contradict this hypothesis. We also discuss SOD1 as a potential therapeutic target for both familial and sporadic ALS.

## Introduction

Amyotrophic lateral sclerosis (ALS) is the most common motor neuron disease and is clinically characterized by the degeneration of motor neurons in the brain and spinal cord, culminating in paralysis and death within 2–5 years. The only available treatment is riluzole, which in the best cases extends survival by only a few months (Glicksman, 2011). In 1993, mutations in the *SOD1* gene encoding Cu,Zn superoxide dismutase-1 were reported as the first genetic link to familial, or inherited, forms of ALS (FALS) (Rosen et al., 1993). Because of the high incidence of *SOD1* mutations, which account for 20–25% of FALS cases, *SOD1* has been one of the most intensely studied genes in the ALS field and continues to be a primary therapeutic target (Bosco and Landers, 2010).

Much of what we understand about the pathomechanisms of ALS is based on *in vivo* studies with transgenic rodent models expressing FALS-linked SOD1 variants. These FALS-SOD1 animal models recapitulate many key features of the human disease, including motor neuron degeneration, paralysis and shortened life-span (Turner and Talbot, 2008). Moreover, FALS-SOD1 animal models reveal the complex nature of this disease, which involves oxidative stress, a loss of proteostasis (i.e., protein aggregation with defective protein clearance), mitochondrial dysfunction, impaired axonal transport, and glutamate excitotoxicity (Rothstein, 2009). While motor neurons are the primary target in ALS, ALS may actually represent a non-cell autonomous disorder for which glia play an active role (Ilieva et al., 2009). Despite decades of research on FALS-SOD1 *in vitro* and *in vivo*, the exact mechanism of SOD1 in ALS pathogenesis remains unknown (Pasinelli and Brown, 2006; Ling et al., 2013). However, as will be discussed throughout this review, a substantial body of literature points to a gain of toxic function for FALS-SOD1 that stems, at least in part, from SOD1 misfolding.

In contrast to FALS, much less is known about the etiology of sporadic ALS (SALS), which accounts for 90% of ALS cases. Pathological aggregates composed of the TAR DNA-binding protein 43 (TDP-43) are detected in CNS tissues for a majority of SALS cases (Neumann et al., 2006), providing strong evidence for an association of misfolded TDP-43 with ALS pathogenesis (Xu, 2012). Mutations in TDP-43 have also been linked to SALS and FALS (Sreedharan et al., 2008), further establishing a role for this protein in disease. Another RNA-binding protein called fused in sarcoma/translocated in liposarcoma (FUS/TLS) has also been linked to FALS and SALS (Kwiatkowski et al., 2009; Vance et al., 2009). Although the association of FUS/TLS with pathological aggregates in SALS has been reported (Deng et al., 2010), this association is not as common as for TDP-43. The inheritable nature of FALS facilitates the identification of causal genes, because many FALS-linked genes, such as *SOD1*, *TDP-43* and *FUS/TLS*, are autosomal dominant and segregate according to Mendelian genetics within an ALS family. In an effort to identify genetic susceptibility factors associated with SALS, several genome-wide association studies (GWAS) have been performed. However, by and large these studies have failed to generate confirmed SALS-susceptibility genes (Bosco and Landers, 2010). More recently, repeat expansions within the genome that are associated with different forms of ALS have been identified. Hexanucleotide repeat expansions in the *C9ORF72* gene were linked to familial frontotemporal lobar degeneration (FTLD)/ALS, FALS as well as to SALS (Dejesus-Hernandez et al., 2011; Renton et al., 2011), making this gene the most common factor in all of ALS. Moreover, repeat expansions in ataxin-2 (*ATXN2*) represent a susceptibility factor in SALS (Elden et al., 2010). Despite these advances in the genetics of SALS, the etiology remains unknown for a majority of SALS cases, likely a reflection of the complex nature of SALS. In fact, SALS may arise from genetic as well as environmental and behavioral factors. Smoking, diet, excessive exercise, injury and exposure to environmental toxins have all been implicated in SALS (D'Amico et al., 2013), although none have been shown to unequivocally cause disease. Although FALS is inheritable and SALS is not, the fact that FALS and SALS are clinically indistinguishable raises the possibility that they do in fact emerge from a common source and/or involve similar toxicity factors.

Recent evidence supports SOD1 as a toxic factor that is common to a subset of both FALS and SALS. This evidence is largely based on the observation that aberrant conformations of WT SOD1, induced by oxidation, demetallation and other altered post-translational modifications, cause WT SOD1 to acquire the same toxic functions that are observed for FALS-associated SOD1 variants (Ezzi et al., 2007; Bosco et al., 2010; Guareschi et al., 2012). Moreover, by employing conformation specific antibodies that are selective for SOD1 only when it is mutated or has altered post-translational modifications, misfolded “mutant-like” WT SOD1 has been detected in human post-mortem tissues from SALS individuals (Bosco et al., 2010; Forsberg et al., 2010, 2011; Pokrishevsky et al., 2012), suggesting that such species are in fact pathogenic. The concept that critical proteins can become pathogenic via both germline mutations and non-Mendelian post-translational modifications is not novel, but rather has strong precedence in neurodegeneration with examples including the α-synuclein (Beyer and Ariza, 2013), tau (Mandelkow et al., 1996) and TDP-43 proteins (Arai et al., 2010). The concept that WT SOD1 could play a role in SALS is controversial, since not all conformation specific antibodies employed to date have detected aberrant WT SOD1 species in SALS (Furukawa, 2012; Ling et al., 2013). However, such a role should be strongly considered and fully explored as it has important therapeutic implications for treating both familial and sporadic forms of ALS.

## Normal properties and cellular functions of SOD1

Long before SOD1 was identified as a causative factor in FALS (Rosen et al., 1993), the anti-oxidizing and catalytic properties of native SOD1 were being investigated (McCord and Fridovich, 1969). While the role of SOD1 as an anti-oxidizing enzyme is well known and accepted, the role of SOD1 as a signaling molecule has been relatively underappreciated. Herein we discuss what is known regarding the normal functions of SOD1 in the context of both anti-oxidation and signaling. The normal physiological properties of SOD1 are discussed in order to establish a foundation for the following sections that describe how both FALS-linked mutations and post-translational modifications alter the structure and function of the protein.

### SOD1: location, activity, and structure

Copper,zinc- superoxide dismutase-1 (SOD1) is a member of the human SOD family of proteins, which also includes SOD2 and SOD3. While all three proteins function as anti-oxidizing enzymes that catalyze the dismutation of superoxide radicals (O_2_•^−^) to hydrogen peroxide (H_2_O_2_), they are distinct proteins with unique characteristics (Zelko et al., 2002). SOD1 is highly abundant, comprising ~1% of total protein in the cell (Pardo et al., 1995), and resides mainly in the cytosol with some degree of localization in the mitochondrial inner membrane space (Fukai and Ushio-Fukai, 2011). The mitochondrion is also home to SOD2, which is localized to the mitochondrial matrix. In contrast to both SOD1 and SOD2, SOD3 is predominately located outside the cell in the extracellular matrix. Other key differences amongst the SOD proteins include their quaternary structures and mechanism of superoxide dismutation: SOD1 is a homodimer while SOD2 and SOD3 are homotetrameric proteins; SOD1 and SOD3 catalyze the dismutation of O_2_•^−^ through the alternate reduction and reoxidation of Cu^2+^, whereas SOD2 utilizes manganese (Mn) as a redox active transition metal for this purpose. The role of SOD in FALS, which will be discussed in detail below, is specific to the SOD1 isoform as there is no compelling evidence supporting the involvement of either SOD2 or SOD3 in FALS pathogenesis (Tomkins et al., 2001).

**Figure 1:**
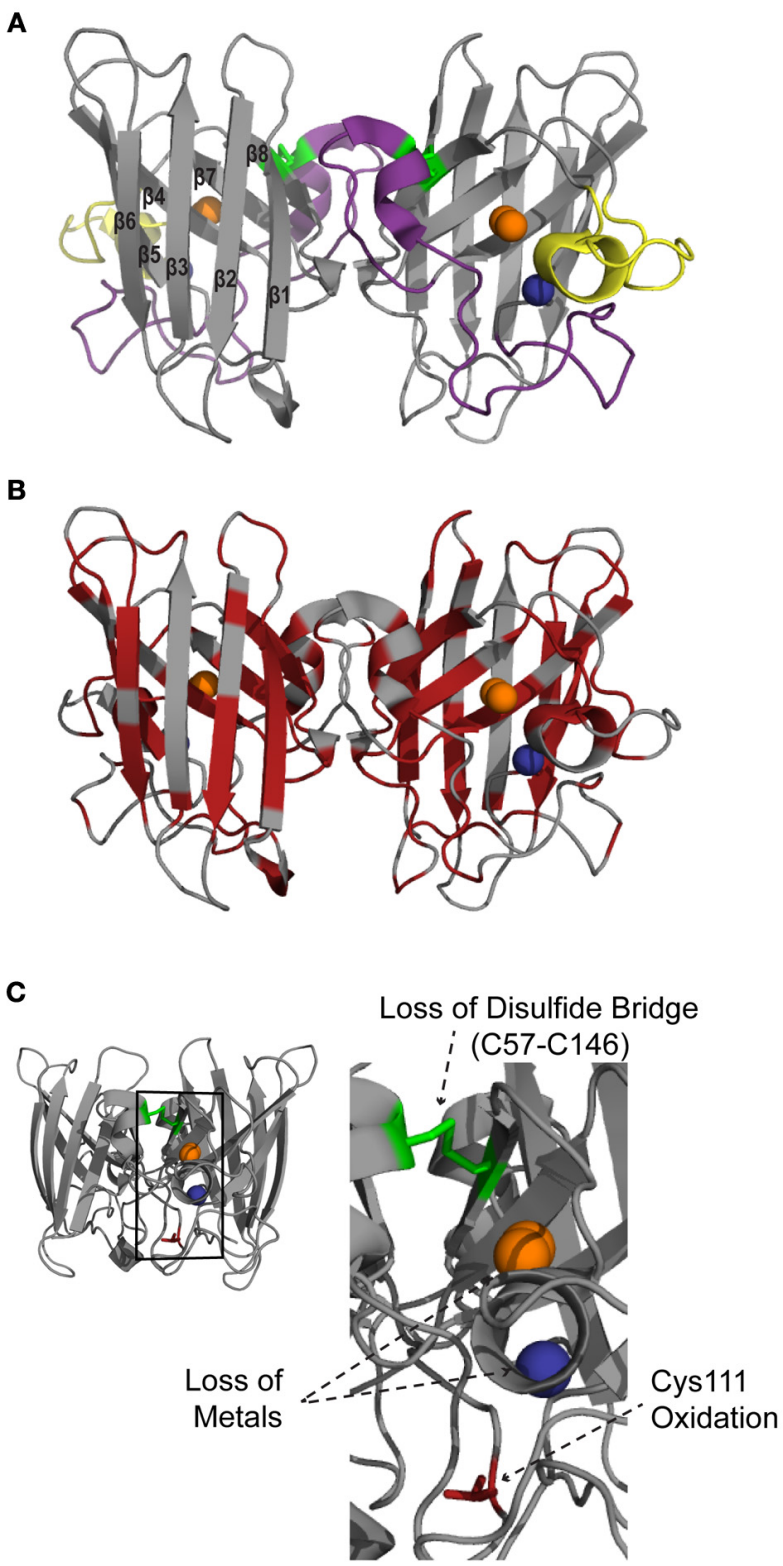
**Structural features of SOD1.** SOD1 is a homodimer with one copper (orange) and one zinc (blue) per subunit (PDB:2C9V). **(A)** SOD1 consists of eight beta sheets that form the beta barrel core. The major functional loops are the zinc binding loop (purple; residues 49–81) and electrostatic loop (yellow; residues 124–139). An intramolecular disulfide bond (C57–C146; green) stabilizes the protein structure. **(B)** Mapping the ALS-linked mutations (red) onto the structure of SOD1 illustrates that these mutations are present throughout the entire protein. **(C)** Alterations to normal post-translational modifications, such as loss of the disulfide bridge (C57–C146; green), demetallation and monomerization (not shown), as well as aberrant modifications such as oxidation of amino acid side chains (shown for Cys 111; red), cause WT SOD1 to misfold.

While coordination of copper to SOD1 is required for dismutation of O_2_•^−^, other post-translational modifications, such as Zn^2+^ coordination (Kayatekin et al., 2008) and disulfide oxidation, help create a mature and structurally stable protein. The 32 kDa homodimeric SOD1 protein adopts an eight-stranded Greek key beta-barrel structural motif (Figure 1A). Two functional loops are present in SOD1: the electrostatic loop that guides superoxide into the redox active site where Cu^2+^ is located and the zinc-binding loop. All tolled, each SOD1 molecule coordinates two copper and two zinc atoms, one of each per subunit. A unique functional feature of SOD1 is the presence of an intrasubunit disulfide bond between Cys57 and Cys146 (C57–C146), which is unusual for proteins that reside in the highly reducing environment of the cytosol. Both copper coordination and formation of C57-C146 is facilitated by the cytosolic copper carrier protein CCS (copper chaperone for SOD1) (Furukawa et al., 2004; Seetharaman et al., 2009). A recent study utilizing both electrospray ionization mass spectrometry (ESI-MS) and nuclear magnetic resonance (NMR) spectroscopy support a step-wise model for SOD1 maturation: (i) SOD1 is loaded with Zn, (ii) heterodimerization between SOD1 and CCS, (iii) Cu is transferred from CCS to SOD1, (iv) C57–C146 is formed, and (v) SOD1 homodimerization (Banci et al., 2012). Together these post-translational modifications produce a highly stable protein, as evidenced by a high melting temperature (T_m_) of ~92°C and resistance to denaturation in both 6 M GdmCl and 4% SDS (Forman and Fridovich, 1973; Bartnikas and Gitlin, 2003). Demetalation of SOD1 and/or reduction of C57–C146 destabilizes the protein and drastically decreases the melting temperature (Forman and Fridovich, 1973; Furukawa and O'Halloran, 2005). As will be discussed below, these post-translational modifications are compromised by both FALS-linked mutations and oxidation, which in turn destabilize SOD1 in the context of disease.

### SOD1 in signal transduction

**Figure 2:**
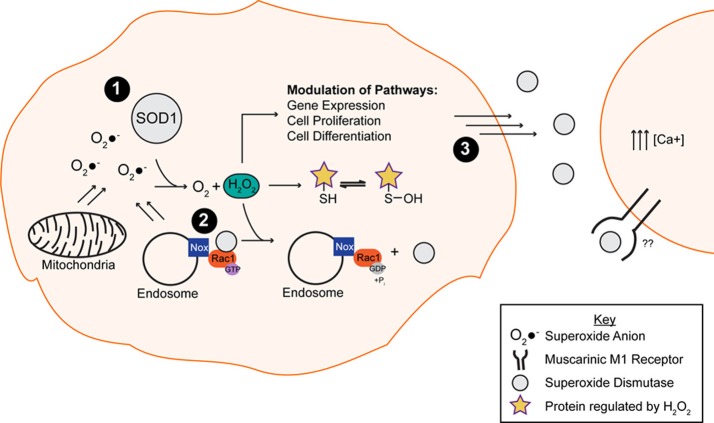
**The putative normal function of the native WT SOD1 protein.** (**1**) SOD1 is responsible for converting the toxic superoxide anion (O_2_•^−^) into oxygen (O_2_) and hydrogen peroxide (H_2_O_2_), the latter of which is involved in the modulation of multiple pathways through oxidation of exposed thiols. (**2**) SOD1 binds and stabilizes Rac1 in its active, GTP bound state, resulting in Nox2 (Nox) activation and superoxide production. The hydrogen peroxide by-product of the dismutase reaction of SOD1 and superoxide anion promotes the disassociation of SOD1 from the Rac1 complex, resulting in Nox inactivation. **(3)** Secreted SOD1 plays a role in extracellular signaling processes. The presence of extracellular SOD1 leads to an increase in intracellular calcium via a mechanism involving the phospholipase C/protein kinase C pathway.

The physiological relevance of SOD1 catalysis extends beyond oxidative stress protection. In fact, SOD1 catalysis plays a key role in signal transduction, a function that is largely underappreciated compared to its role as an anti-oxidizing enzyme (Figure 2). For instance, H_2_O_2_ generated by SOD1 can reversibly and specifically react with proteins, generally by oxidizing Cys residues. Cys oxidation in turn alters the biochemical and functional properties of those proteins in a redox dependent manner (Georgiou, 2002). A variety of signal transduction pathways are modulated by H_2_O_2_, including but not limited to gene expression, cell proliferation, differentiation and death (Rhee, 2006; Brown and Griendling, 2009). NADPH oxygenases (Nox) function as upstream regulators of these signal transduction pathways through the production of O_2_•^−^, which is either converted to H_2_O_2_ spontaneously or catalytically by SOD1. SOD1 comes into close proximity with Nox2-derived O_2_•^−^ at the surface of endosomes in response to proinflammatory cytokines (Harraz et al., 2008). A report by Harraz et al. (2008) demonstrated that SOD1 not only acts downstream of Nox2 but can also modulate Nox function through an interaction with Rac1. SOD1 directly binds and stabilizes the active form of Rac1 in its GTP-bound state, leading to Nox2 activation and O_2_•^−^ production. Interestingly, H_2_O_2_ generated by SOD1 serves as a negative feedback of Nox2 activity: H_2_O_2_ induces the dissociation of the SOD1/Rac1 complex, thereby inactivating Rac1 and Nox2 (Harraz et al., 2008). The mechanism for how H_2_O_2_ disrupts the interaction between SOD1 and Rac1 has not been elucidated. One possibility is that the H_2_O_2_ generated by SOD1, which is in close proximity to Rac1, oxidatively modifies Cys residues within Rac1 in such a way that disrupts the SOD1/Rac1 binding interaction.

Another example of SOD1 redox-sensing and signaling activity is in the context of respiratory repression, which occurs during aerobic fermentation in proliferating cells, including in the context of some cancers. In *Saccharomyces cerevisiae*, a loss of SOD1 activity impairs respiratory repression (Sehati et al., 2011). SOD1 was shown to modulate respiratory repression through binding and stabilizing Yck1p and Yck2p, two casein kinase 1-gamma homologs in yeast that inhibit respiration (Reddi and Culotta, 2013). The authors speculate that SOD1 stabilizes Yck1p/Yck2p through the action of the reaction product H_2_O_2_, where oxidative modification of lysine residues within Yck1p/Yck2p by H_2_O_2_ prevents their ubiquitination and degradation by the ubiquitin-proteasome system. Therefore, both the Rac1/Nox2 and Yck1p/Yck2p interactions with SOD1 demonstrate that the catalytic action of SOD1 can direct the modification and activity of specific protein substrates. In this manner, SOD1 catalyzes a “molecular redox switch” that ultimately controls protein function and signaling, much like phosphorylation. While the catalytic activity of SOD1 is required for these interactions, it is not simply to remove O_2_•^−^ from circulation, but rather to modulate signaling pathways in a redox sensitive manner. One can imagine that redox signaling needs to be regulated, as an excess of either O_2_•^−^or H_2_O_2_ would have deleterious effects on the cell. The levels of H_2_O_2_ in the cell are further controlled by the antioxidant enzymes catalase, peroxiredoxins and glutathione peroxidases (Fukai and Ushio-Fukai, 2011), which convert H_2_O_2_ into water and oxygen.

Extracellular SOD1 has also been shown to play a role in signaling. Although SOD1 is predominately localized to the cytoplasm, multiple reports have demonstrated that SOD1 is secreted (Mondola et al., 1996, 1998, 2003; Cimini et al., 2002; Turner et al., 2005). The presence of extracellular SOD1 can in turn increase intracellular calcium levels (Mondola et al., 2004), a phenomena shown to have neuroprotective effects on cerebellar granular neurons exposed to a dopaminergic toxin (Polazzi et al., 2012). This increase in intracellular calcium results from SOD1 activating the phospholipase C/protein kinase C pathway, a pathway implicated in calcium homeostasis (Mondola et al., 2004) through a mechanism involving signal transduction of the muscarinic acetylcholine M1 receptor (M1) (Damiano et al., 2013). Moreover, M1 activation in response to extracellular SOD1 jumpstarts downstream pathways such as the extracellular regulated protein kinase (ERK 1/2) and the Akt signaling cascades (Damiano et al., 2013). Unlike the previous SOD1 signaling pathways discussed above, the activation of the M1 receptor does not appear to be dependent upon the production of O_2_•^−^, as the ROS scavenger N-acetylcysteine did not alter the signaling effect of SOD1 in this context.

## SOD1 misfolding: lessons from familial ALS

For the past 20 years, mutant-SOD1 has been the most intensely studied molecule in the ALS field. Studies in animal and cell culture models, as well as extensive biochemical and biophysical analyses of recombinant mutant-SOD1 proteins have collectively revealed a gain-of-toxic mechanism for mutant-SOD1 in ALS that is linked to its propensity to misfold. Aberrantly modified WT SOD1 adapts a conformation and toxic nature much like FALS-linked SOD1 mutants, and is therefore proposed as a pathogenic factor in SALS. Below we introduce what is known about the structure and aberrant properties of FALS-SOD1.

### FALS-linked SOD1 mutations: effect on structure and conformation

Currently, 171 mutations have been identified within SOD1 that are linked to ALS (http://alsod.iop.kcl.ac.uk/) (Abel et al., 2012). Approximately 20–25% of FALS cases and 6% of all ALS cases are caused by mutations in *SOD1* (Pasinelli and Brown, 2006). The majority of these mutations (>80%) result in amino acid substitutions while the remaining lesions are a combination of insertions, polymorphisms, and deletions. FALS-linked mutations are not localized to one portion of SOD1, but rather span the entire protein (Figure 1B). Moreover, relatively conservative amino acid substitutions within SOD1 can cause ALS, suggesting that even minor alterations severely affect SOD1 structure and/or function. Much effort has been focused on determining the common “toxic” feature within SOD1 that is induced by all of these ALS-linked mutations. Except for the mutations that directly interfere with metal coordination, as copper coordination is required for catalytic activity, many ALS-linked mutations have no effect on SOD1 dismutase activity (Borchelt et al., 1994; Bowling et al., 1995; Hayward et al., 2002; Potter and Valentine, 2003). The effect of ALS-linked mutations on SOD1 signaling activity remains largely unexplored. However, in the context of Nox2 signaling, FALS-linked SOD1 mutations were shown to alter the SOD1/Rac1 interaction, resulting in persistent activation of Nox2 and abnormally high levels of ROS (Harraz et al., 2008).

**Figure 3:**
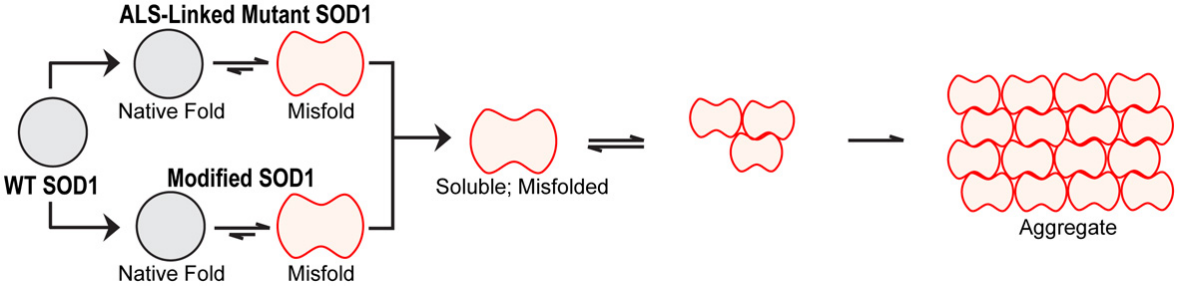
**Misfolded vs. aggregated SOD1.** Misfolded SOD1 results from both ALS-linked mutations and aberrant post-translational modifications. Throughout the review, the term “misfolding” refers to the structural loosening of SOD1 due to a mutation and/or altered post-translational modification within a soluble form of the protein. Misfolded, soluble SOD1 can engage in aberrant protein-interactions and acquire new toxic functions (illustrated in Figure 5). Misfolded SOD1 can also assemble into aggregates. In this review, “aggregates” refer to those insoluble species characteristic of end-stage pathological inclusions present in human post-mortem CNS tissues.

A general feature of FALS-linked SOD1 mutants is that they are destabilized and exhibit reductions in T_m_ relative to WT SOD1 (Stathopulos et al., 2006; Vassall et al., 2006). In fact, FALS-linked SOD1 mutants are further destabilized when normal post-translational modifications such as Cu/Zn coordination and C57–C146 oxidation are impaired (Furukawa and O'Halloran, 2005; Rodriguez et al., 2005; Kayatekin et al., 2010; Svensson et al., 2010), demonstrating interconnection between normal post-translational modifications and the stability of SOD1. Additionally, mutant SOD1 exhibits an altered tertiary structure, evidenced by enhanced hydrophobicity compared to WT SOD1 (Tiwari et al., 2005; Munch and Bertolotti, 2010). While X-ray crystallography has failed to reveal significant structural differences between WT- and mutant-SOD1 proteins, (Hart et al., 1998; Elam et al., 2003; Hough et al., 2004; Cao et al., 2008; Galaleldeen et al., 2009), solution-based structural studies indicate that FALS-linked mutations induce some degree of SOD1 unfolding, or “misfolding” (Figure 3), within the electrostatic and/or zinc loops (Figure 1). For example, NMR relaxation experiments reveal an overall increase in protein dynamics for SOD1 G93A compared to WT SOD1 (Shipp et al., 2003). The region proximal to the G93A mutation, termed the “β-barrel plug,” as well as residues within the zinc-binding loop exhibited the greatest increase in mobility, whereas the dynamics within the electrostatic loop were comparable between SOD1 G93A and WT (Shipp et al., 2003). Similarly, β-strands 3 and 4, which contain the β-barrel plug, displayed high deuterium exchange rates as assessed by mass spectrometry for several demetallated mutant forms of SOD1 (Durazo et al., 2009). In a separate study using similar methodology but with metallated forms of SOD1, only 1 out of the 13 mutants exhibited relatively high deuterium exchange rates in the β-barrel plug (Molnar et al., 2009). However, this study detected a 10–31% increase in deuterium exchange within the electrostatic loop for 7 out of 8 “WT-like” ALS-linked SOD1 mutants (i.e., mutant-SOD1 proteins that coordinate Cu and Zn), including A4V. Deuterium exchange increased within both the electrostatic and zinc binding loops for those SOD1 mutants with impaired metal binding (Molnar et al., 2009).

**Figure 4:**
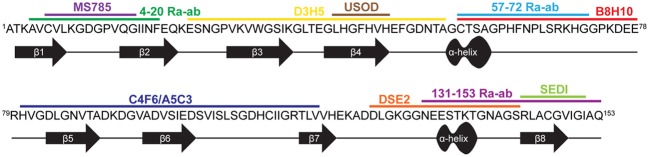
**Conformation specific antibodies highlight regions exposed in SOD1 upon misfolding.** The primary sequence for WT SOD1 is shown with the secondary structure displayed below. The binding regions of 11 antibodies specific for misfolded SOD1 are depicted above the sequence (see Table 1 for antibody details).

The generation of conformation specific antibodies has also provided important insights into the misfolded nature of FALS-linked mutant-SOD1 proteins. Most conformation specific antibodies reported to date are selectively reactive for mutant SOD1 over WT SOD1, consistent with the notion that mutations induce some degree of misfolding that exposes linear sequences or conformational epitopes that are otherwise buried in the intact, native protein (Rakhit et al., 2007; Urushitani et al., 2007; Liu et al., 2009; Bosco et al., 2010; Forsberg et al., 2010; Gros-Louis et al., 2010; Grad et al., 2011; Brotherton et al., 2012; Fujisawa et al., 2012; Broering et al., 2013) (Figure 4, Table 1). For example, the conformation specific antibodies B8H10, D3H5, and A5C3 that were generated against apo-SOD1 G93A do not immunoprecipitate WT SOD1, but rather exhibit differential reactivity for various mutant-SOD1 proteins (G93A, G37R, G85R, G127X, and D90A) from spinal cord lysates derived from the respective ALS mouse models. That B8H10, D3H5, and A5C3 become reactive for denatured WT SOD1 is consistent with a linear epitope within natively folded WT SOD1 that is exposed only upon SOD1 unfolding (Gros-Louis et al., 2010). The C4F6 antibody is also selective for FALS-linked SOD1 mutants G37R, G85R, G93A (Urushitani et al., 2007) A4V (Brotherton et al., 2012) over WT SOD1 when these proteins are in their native state but exhibits weak reactivity for denatured SOD1 proteins, suggesting that this antibody recognizes a conformational epitope (Bosco et al., 2010). Interestingly, both D3H5 (Gros-Louis et al., 2010) and C4F6 (Brotherton et al., 2012) immunoreactivity for SOD1 G93A directly correlates with disease progression in the transgenic SOD1 G93A ALS mouse model, indicating that these antibodies report on the presence of “toxic,” misfolded forms of SOD1 G93A.

**Table 1:** **Conformation specific antibodies recognize regions of SOD1 exposed upon misfolding**.

**Antibody**	**SOD1 epitope (aa)**	**Reactive for FALS SOD1**	**Reactive for SALS SOD1**		**Therapeutic benefits**	**Reference**
			***Patient tissue***	***Modified SOD1***		
B8H10^e^	57–78^a^	+	nd^b^	nd	nd	Gros-Louis et al., 2010; Pickles et al., 2013
C4F6^e^	80–118	+	+	+ (ox)	nd	Urushitani et al., 2006; Bosco et al., 2010; Prudencio and Borchelt, 2011; Brotherton et al., 2012; Pickles et al., 2013
MS785	6–16	+	nd	+^c^	nd	Fujisawa et al., 2012
D3H5	24–55^a^	+	nd	+ (apo)	+	Gros-Louis et al., 2010
A5C3^e^	80–118^a^	+	nd	nd	−	Gros-Louis et al., 2010
SEDI	143–151	+	−	+ (ox)	+^d^	Rakhit et al., 2007; Liu et al., 2009, 2012; Kerman et al., 2010; Prudencio and Borchelt, 2011; Mulligan et al., 2012
USOD	42–48	+	−	− (ox)	nd	Kerman et al., 2010; Mulligan et al., 2012
DSE2 (3H1)	125–142	+	+	nd	nd	Vande Velde et al., 2008; Grad et al., 2011; Pokrishevsky et al., 2012
4–20Ra-ab	4–20	+	+	nd	nd	Jonsson et al., 2004; Forsberg et al., 2010
57–72Ra-ab	57–72	+	+	nd	nd	Stewart et al., 2006; Forsberg et al., 2010, 2011
131–153Ra-ab	131–153	+	+	nd	nd	Jonsson et al., 2004; Forsberg et al., 2010, 2011

Misfolded SOD1-specific antibodies highlight regions in SOD1 that become exposed as a result of misfolding.

a Determined using similar methodology as in C4F6 epitope mapping (Bosco et al., 2010) (unpublished data, Bosco DA).

b nd, Not determined.

c Serum starvation in cultured cells results in SOD1 reactivity with antibody.

d Benefits observed from active immunization with SOD1 peptide recognized by SEDI.

e Commercially Available.

In contrast to the aforementioned antibodies, for which the epitope within SOD1 remains unknown, several conformation specific antibodies have been generated against sequences that are predicted to become exposed only upon SOD1 misfolding (Rakhit et al., 2007; Grad et al., 2011; Fujisawa et al., 2012). One such antibody, MS785, targets amino acids 6-16 at the N-terminus of SOD1. This portion of mutant SOD1 is implicated in binding Derlin-1, a protein involved with ER associated degradation, and is thus referred to as the Derlin-1 binding region (DBR). Fujisawa et al., demonstrated through immunoprecipitation experiments that mutant-SOD1 associates with Derlin-1 when coexpressed in HEK cells. Their comprehensive immunoprecipitation strategy assessed the interaction between Derlin-1 and 132 ALS-linked SOD1 mutants, of which 124 were found to associate with Derlin-1. Further, MS785 selectively immunoprecipitated SOD1 from B-lymphocytes derived from all 14 SOD1 positive ALS cases examined but not from 11 healthy controls (Fujisawa et al., 2012). An alternative antibody called SEDI was created against an epitope at the opposite end of the SOD1 molecule. SEDI stands for SOD1 Exposed Dimer Interface and was raised against the amino acids 143–151 located at the dimer interface in SOD1 (Rakhit et al., 2007). SEDI reactivity is specific for mutant SOD1 over native WT SOD1 in the context of transgenic mouse tissue [G37R, G93A, and G85R (Rakhit et al., 2007)] and human post-mortem tissues harboring SOD1 mutations [A4V (Rakhit et al., 2007), A4T, V14M, ΔG27/P28, and I113T (Liu et al., 2009)]. Therefore, MS785 and SEDI antibodies report on misfolding events within the N- and C-termini, respectively, that are induced by ALS-linked mutations.

A direct consequence of mutation-induced misfolding of SOD1 is aggregation, which refers to the irreversible assembly of misfolded SOD1 species into an insoluble structure (Figure 3). SOD1 aggregation has been extensively investigated *in vivo*, both in ALS human post-mortem tissues and in mutant-SOD1 transgenic mice. The enhanced aggregation propensities of FALS-linked SOD1 mutants have also been comprehensively examined in cell culture and in other *in vitro* assays. We refer the reader to many excellent reviews and original works that detail current models of mutant-SOD1 aggregation (Durham et al., 1997; Bruijn et al., 1998; Johnston et al., 2000; Stathopulos et al., 2003; Furukawa et al., 2006; Wang et al., 2008; Prudencio et al., 2009) Review: (Chattopadhyay and Valentine, 2009; Turner and Talbot, 2008).

Although it remains unclear whether SOD1 aggregation is a causative or protective factor in disease progression, several recent reports demonstrate that misfolded SOD1 species can spread from cell to cell in a prion-like fashion (Grad et al., 2011; Munch et al., 2011; Sundaramoorthy et al., 2013). Munch et al demonstrated that the uptake of aggregated ALS-linked SOD1 mutants in cultured neuronal cells seeded aggregation of endogenous mutant-SOD1. These endogenous SOD1 aggregates persisted well after (>30 days) the original aggregates dissipated from cell division, consistent with a prion-like propagation of aggregated SOD1 (Munch et al., 2011). More recently, uptake of both misfolded mutant-SOD1 as well as aggregated mutant-SOD1 was shown to induce aggregation of the native WT SOD1 protein (Sundaramoorthy et al., 2013). This latter report demonstrates how misfolded SOD1 can alter the conformation of otherwise normally folded SOD1, however, it remains to be determined whether this spreading of misfolded and aggregated SOD1 directly impacts ALS pathogenesis *in vivo* (Guest et al., 2011).

### Misfolded FALS-linked SOD1 exerts a gain of toxic function in ALS

Studies using transgenic rodent models have generated the majority of data pointing to a gain of toxic mechanism for mutant-SOD1 in FALS (Turner and Talbot, 2008). Several transgenic mouse models have been engineered to overexpress ALS-linked SOD1 mutants. Mutant-SOD1 transgenic mice develop an ALS-like phenotype that includes motor neuron degeneration, neuroinflammation, severe paralysis and premature death (Gurney et al., 1994; Dal Canto and Gurney, 1995; Wong et al., 1995; Bruijn et al., 1997; Dal Canto and Gurney, 1997). In addition, cytosolic SOD1-containing ubiquitinated aggregates are detected within CNS tissues from these mice, recapitulating the pathological features of the human disease (Gurney et al., 1994; Dal Canto and Gurney, 1995; Bruijn et al., 1997; Dal Canto and Gurney, 1997; Watanabe et al., 2001). These mice express endogenous murine SOD1, and yet develop motor neuron disease upon expression of exogenous human mutant-SOD1, providing evidence that SOD1 mutations lead to a gain of toxic function. Further, SOD1-deficient mice develop normally with no overt signs of neurodegeneration (Reaume et al., 1996). However, it is noted that SOD1 null mice are more susceptible to axonal (Reaume et al., 1996) and ischemic brain (Kondo et al., 1997) injuries, and therefore a complete loss of SOD1 may be disadvantageous, especially in the context of disease (van Blitterswijk et al., 2011).

Because the aforementioned SOD1 pathological aggregates are a downstream consequence of SOD1 misfolding, the presence of such aggregates in human post-mortem tissues and ALS-mice argues for a role of misfolded SOD1 in disease. This notion is supported by immunization strategies that both target misfolded SOD1 species and have therapeutic outcome in ALS mice. A passive immunization strategy with the D3H5 antibody that specifically reacts with misfolded SOD1 extended survival in SOD1 G93A transgenic mice (Gros-Louis et al., 2010)(Figure 4, Table 1). A greater therapeutic impact was shown in active immunization trials with the SOD1 G37R transgenic mouse model, using both recombinant apo-SOD1 G93A (Urushitani et al., 2007) and the SEDI (SOD1 exposed dimer interface) peptide (Liu et al., 2012) as immunogens. Use of SEDI increased survival and delayed disease onset to a greater extent than the full-length SOD1 immunogen, likely due to specific targeting of a misfolded toxic epitope within mutant-SOD1 (Liu et al., 2009, 2012). While these studies provide a direct correlation between misfolded SOD1 species and disease in mice, a survival benefit was not realized using humanized SOD1 antibodies in SOD1 transgenic mice (Broering et al., 2013). Going forward, immunotherapeutic strategies in humans may require the specific targeting of regions within SOD1 that are only exposed upon misfolding. Moreover, these regions should mediate some toxic effect *in vivo*, so that antibodies have the potential to neutralize or otherwise block that “toxic epitope.” The latter criterion is important in light of the fact that not all mutant-specific antibodies have produced a therapeutic outcome in SOD1 transgenic mice (Gros-Louis et al., 2010).

The immunization studies described above demonstrate that misfolded SOD1 can impact the disease course in ALS mice. What evidence supports a toxic role of misfolded and/or aggregated SOD1 in the human disease? Wang et al., found an inverse correlation between SOD1 aggregation propensity and disease duration in human ALS cases (i.e., mutants that are more aggregation prone are associated with cases that exhibit relatively short survival) using the Chiti-Dobson equation (Wang et al., 2008). A complementary study determined the relative aggregation propensities of 30 SOD1 mutants in a cell culture assay and also reported an indirect correlation between SOD1 aggregation propensity and disease duration (Prudencio et al., 2009). However, this correlation was not statistically significant, possibly due to the limited number of SOD1 mutants that could be included in this type of analysis that utilizes patient data (Prudencio et al., 2009). Nonetheless, these studies implicate misfolded SOD1 as a factor in human ALS pathogenesis.

### Toxic, soluble misfolded FALS-SOD1 species in disease

**Figure 5:**
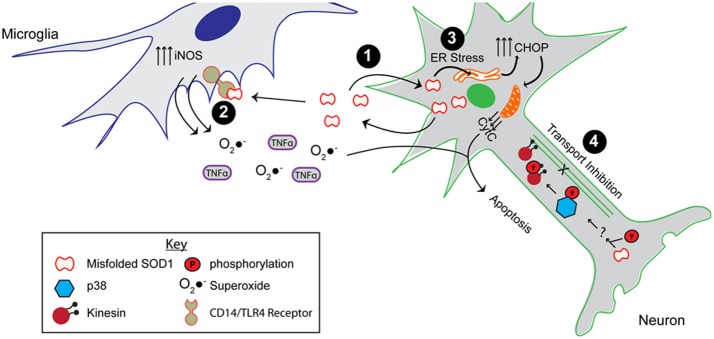
**The toxic properties shared by ALS-linked mutant SOD1 and modified WT SOD1.** As shown, misfolded SOD1, resulting from mutations or aberrant post-translational modifications, causes the protein to engage in aberrant interactions. **(1)** Misfolded SOD1 is both secreted, possibly through chromogranins, and taken up from the extracellular environment. **(2)** Extracellular misfolded SOD1 activates microglia by binding to the CD14/TLR4 receptor, thereby elevating nitric oxide synthase (iNOS) activity as well as secretion of superoxide anion (O_2_•^−^) and proinflammatory cytokines (e.g., TNFα). **(3)** Expression and uptake of misfolded SOD1 leads to ER stress, which elevates the pro-apoptotic CHOP protein and induces mitochondrial damage. **(4)** The presence of misfolded SOD1 in the axon results in axonal transport inhibition through a mechanism involving the phosphorylation of p38 MAPK and the kinesin motor. All of these aberrant functions compromise the integrity of the motor neuron, and potentially contribute to both FALS and SALS pathogenesis.

While end-stage pathological aggregates composed of insoluble mutant-SOD1 are detected in ALS-mouse models and in the human disease, emerging evidence suggests that the toxic SOD1 species is in fact a misfolded, soluble form of the protein (Figure 3). One can imagine that an aggregated, insoluble form of SOD1, much like what is found at end stage of disease, is isolated and unable to diffuse within the cell. Conversely, a non-aggregated misfolded form of SOD1 is soluble, accessible, and able to engage in aberrant interactions, thereby enabling a gain of toxic function. To enumerate all of the aberrant functions and interactions that have been observed for mutant-SOD1 is beyond the scope of this review and therefore we refer the reader to excellent reviews that cover this topic (Cleveland and Rothstein, 2001; Bruijn et al., 2004; Pasinelli and Brown, 2006; Joyce et al., 2011). Herein, we will focus on those gain-of-toxic functions for FALS-linked mutants that are thought to involve misfolded, soluble SOD1 and that may also have relevance to sporadic ALS (Figure 5).

Support for a soluble misfolded form of SOD1 comes from studies in both SOD1 animal models and cell culture. Zetterstrom et al., identified an accumulation of soluble misfolded SOD1 that was enriched in the spinal cord of SOD1 G93A transgenic mice. The fraction of soluble misfolded SOD1 was quantified with hydrophobic interaction chromatography (HIC) (Zetterstrom et al., 2007). Interestingly, not only is the solubility of FALS-linked SOD1 variants enhanced upon heterodimerization with WT SOD1, but the toxicity of these variants is also enhanced, consistent with the toxic SOD1 species being soluble-misfolded rather than insoluble-aggregated (Witan et al., 2008, 2009). In CHO cells, the fraction of soluble SOD1 was found to correlate with cellular toxicity (Brotherton et al., 2013). This cellular toxicity was attenuated upon expression of exogenous hsp70 (Brotherton et al., 2013), a chaperone that refolds misfolded proteins, although it is not clear whether this is due solely to a reduced load of misfolded SOD1 or whether the anti-apoptotic function of hsp70 also contributed to this effect (Beckmann et al., 1990; Beere et al., 2000; Luders et al., 2000).

Misfolded mutant SOD1 can have downstream effects both outside and inside the cell. Although WT SOD1 is known to be secreted normally (Mondola et al., 1996, 1998, 2003; Cimini et al., 2002; Turner et al., 2005; Urushitani et al., 2006; Santillo et al., 2007), Urushitani et al. demonstrated that mutant-SOD1 can be secreted by an alternate pathway that involves the secretory chromogranin proteins (Urushitani et al., 2006). In contrast, Turner et al., demonstrated an impaired secretion of mutant SOD1 compared to WT SOD1 (Turner et al., 2005). The discrepancy between these studies may de due to the different cell types employed. Nonetheless, both studies are consistent with a dysregulation of mutant-SOD1 secretion compared to native WT SOD1. Once secreted into the extracellular space, mutant-SOD1 activates microglia though a mechanism that involves binding to the CD14/TLR receptor (Zhao et al., 2010), resulting in a typical proinflammatory response (i.e., increased levels of TNFα and IL-1β). This mode of microglia activation leads to motor neuron death (Urushitani et al., 2006; Zhao et al., 2010).

Inside the cell, mutant SOD1 can induce stress in the context of various pathways. For example, primary neurons derived from SOD1 G93A transgenic mice, as well as cell culture models of mutant-SOD1, exhibit signs of ER stress including spliced XBP1 and induction of CHOP (Nishitoh et al., 2008; Prell et al., 2012). ER stress has been shown to be a major player in ALS pathogenesis [reviewed in Kanekura et al. (2009), Matus et al. (2011)]. It should be noted that endogenous expression of mutant-SOD1 is not required for activation of the ER stress response pathway. Uptake of extracellular SOD1 G93A is sufficient to induce ER stress and neuronal toxicity (Sundaramoorthy et al., 2013). Thus, the combination of mutant-SOD1 uptake and intracellular expression in neurons could exacerbate ER stress, and tilt the scale from the UPR coping mechanism toward apoptosis *in vivo*. Mitochondria represent an additional intracellular compartment that is affected by mutant-SOD1 (Pickles et al., 2013). Recent evidence indicates that mutant-SOD1 directly interacts with Bcl-2, leading to exposure of the toxic BH3 domain, which in turn causes mitochondrial damage (Pasinelli et al., 2004; Pedrini et al., 2010). Interestingly, enhanced exposure of the BH3 domain has been detected in FALS SOD1 A4V patient spinal cord homogenates, and increases as a function of disease in spinal cords of SOD1 G93A transgenic mice (Pedrini et al., 2010). Finally, much evidence suggests that mutant-SOD1 impairs the process of axonal transport in different model systems (Morfini et al., 2009). In studies of fast axonal transport (FAT) performed in squid axoplasm, there is compelling evidence that it is a soluble misfolded form of mutant-SOD1 that inhibits transport in the anterograde direction (Morfini et al., 2009, 2013; Song et al., 2013). Moreover, these studies were extended to explore the role of WT SOD1 in SALS, which is the topic of the following section.

## Misfolded wild-type SOD1: implications for SALS

Since the discovery of FALS-linked mutations in SOD1 (Rosen et al., 1993), there has been speculation that the wild-type version of the protein could play a role in sporadic ALS (Selverstone Valentine et al., 2005; Kabashi et al., 2007). It has become increasingly clear that alterations of the normal post-translational modifications of SOD1 and/or introduction of aberrant modifications in WT SOD1 cause this otherwise stable protein to misfold and adapt properties similar to FALS-linked SOD1 mutants (Figure 1C). Until recently, however, there was a lack of direct evidence for aberrant forms of WT SOD1 in SALS. This is probably because the field lacked the appropriate tools and methodologies to detect such species. Below we describe modifications of WT SOD1 that cause this protein to become misfolded and “toxic.” We also present evidence, largely from the use of conformation specific antibodies that recognize misfolded SOD1 species, that support or contradict the hypothesis that WT SOD1 plays a role in sporadic ALS.

### Loss of native SOD1 post-translational modifications leads to WT SOD1 misfolding

As discussed above, coordination of Zn, oxidation of the C57–C146 intrasubunit disulfide bond and homodimerization of SOD1 contribute to the structural stability of the molecule. A loss in integrity of any one of these normal post-translational modifications compromises the stability of WT SOD1 and contributes to its misfolding and aggregation (Figures 1C, 3). For example, a reduction of WT SOD1 stability upon demetallation is a widely documented observation (Stathopulos et al., 2003; Lynch et al., 2004; Furukawa and O'Halloran, 2005; Ding and Dokholyan, 2008). Interestingly, demetallation of WT SOD1 induces similar conformational perturbations within the zinc binding and the electrostatic loops (loops IV and VII, respectively, Figure 1) as do FALS-linked SOD1 mutations (Strange et al., 2003, 2007; Ding and Dokholyan, 2008; Durazo et al., 2009; Molnar et al., 2009). A direct comparison of NMR backbone chemical shifts for apo dimeric WT SOD1 vs. holo dimeric WT SOD1 revealed the largest structural variations within the electrostatic loop (Banci et al., 2009). These structural alterations were accompanied by pronounced changes in backbone dynamics that further support demetallation-induced misfolding (Banci et al., 2009). Molecular dynamics (Ding and Dokholyan, 2008) and H/D exchange by mass spectrometry (Durazo et al., 2009) also report enhanced flexibility within the aforementioned loops of apo WT SOD1 compared to holo SOD1, but in addition these studies also detect misfolding within the beta barrel of SOD1 upon demetallation (Ding and Dokholyan, 2008; Durazo et al., 2009). Furthermore, the misfolding of WT SOD1 induced by demetallation leads to aggregation of the protein (Banci et al., 2007), which may be driven by the exposure of hydrophobic regions within misfolded SOD1 that are otherwise buried in the native protein (Tiwari et al., 2009).

In addition to demetallation, reduction of the C57–C146 disulfide bond also has a destabilizing effect on WT SOD1 that can lead to protein misfolding and aggregation in a manner similar to FALS-linked mutant SOD1 (Figure 3) (Furukawa et al., 2008; Chan et al., 2013). Several studies have demonstrated that a reduction of C57–C146 in apo-SOD1 shifts the monomer-dimer equilibrium toward the monomeric state. Addition of zinc or oxidation of C57–C146 shifts the equilibrium back to the dimeric state, demonstrating interdependence between Zn coordination, C57–C146 bond integrity, and dimerization on the structural stability of SOD1 (Arnesano et al., 2004; Lindberg et al., 2004; Hornberg et al., 2007).

### Introduction of aberrant post-translational modifications induce WT SOD1 misfolding

In addition to alterations of the normal post-translational modifications of SOD1, WT SOD1 misfolding and aggregation can be induced by the formation of aberrant modifications. Oxidation of SOD1 side chains represents one such modification that has been postulated to play a significant causal role in both FALS and SALS (Kabashi et al., 2007). In the context of WT SOD1, which has relevance to SALS, metal catalyzed oxidation with CuCl_2_ and ascorbic acid leads to oxidative modification of SOD1 histidine residues and subsequent SOD1 aggregation (Rakhit et al., 2002, 2004). This mode of oxidation-induced SOD1 aggregation proceeds by way of SOD1 dimer dissociation/monomer formation, demonstrating interdependence between aberrant and normal SOD1 post-translational modifications (Rakhit et al., 2004). Cys111 within SOD1 is particularly susceptible to H_2_O_2_ induced oxidation (Figure 1C). Prolonged exposure of SOD1 to H_2_O_2_ results in the irreversible conversion of the Cys111 sulfhydryl group to sulfonic acid (Fujiwara et al., 2007; Bosco et al., 2010; Auclair et al., 2013), which may be detrimental since WT SOD1 oxidized by H_2_O_2_ (hereafter referred to as SOD1ox) exhibits an enhanced propensity to misfold and aggregate (Ezzi et al., 2007; Fujiwara et al., 2007; Bosco et al., 2010; Chen et al., 2012). Interestingly, modifications such as β-mercaptoethanol (Fujiwara et al., 2007), persulfide (de Beus et al., 2004) and cysteinylation (Auclair et al., 2013) on Cys111 protect SOD1 against oxidation and possibly against subsequent misfolding.

### Aberrantly modified WT SOD1 proteins are toxic and mimic FALS-linked SOD1 mutants

The observation that zinc depleted WT SOD1 exerts a toxic effect onto motor neurons by a mechanism involving nitric oxide provided one of the first clues that modified WT SOD1 species are toxic and may contribute to SALS pathogenesis (Estevez et al., 1999; Beckman et al., 2001). More recently, zinc-deficient SOD1 was shown to exhibit a toxic effect related to mitochondrial dysfunction in *Drosophila* (Bahadorani et al., 2013). That metal deficient WT SOD1 can be induced to misfold and exhibit a toxic nature analogous to FALS-linked SOD1 is supported by the immunization trial in ALS mice reported by Takeuchi et al. Low copy SOD1 G93A transgenic mice vaccinated with the apo WT SOD1 immunogen exhibited delayed disease onset and prolonged survival compared to control mice injected with saline/adjuvant, and importantly, to a similar degree as mice vaccinated with apo SOD1 G93A (Takeuchi et al., 2010). This study also implicates WT SOD1 as a viable therapeutic target for SALS.

In recent years, several reports have demonstrated a toxic effect of SOD1ox in the context of ALS relevant pathways and processes (Figure 5). For example, SOD1ox acquires aberrant protein-interactions that are also observed for FALS-linked SOD1 mutants. SOD1ox interacts with the heat shock protein Hsc70 that plays a role in refolding misfolded proteins (Ezzi et al., 2007), the secretory protein chromagranin B that actively secretes misfolded forms of SOD1 (Urushitani et al., 2006; Ezzi et al., 2007), and the anti-apoptotic protein Bcl-2 in a manner that induces mitochondrial damage (Pasinelli et al., 2004; Guareschi et al., 2012). SOD1ox was also shown to mimic FALS-linked SOD1 mutants in the inhibition of anterograde FAT in squid axoplasm. These studies demonstrated that the inhibitory effect of SOD1ox and mutant SOD1 was mediated by activated p38 MAPK, indicating that misfolded SOD1 can trigger kinase-dependent signaling cascades (Morfini et al., 2009; Bosco et al., 2010; Morfini et al., 2013; Song et al., 2013). Extracellular derived WT SOD1 can also induce a toxic effect onto cells. SOD1ox applied to cell culture media activates immortalized microglia (Ezzi et al., 2007), which in turn may cause motor neuron death (Ezzi et al., 2007; Zhao et al., 2010). Furthermore, uptake of aggregated WT SOD1 species by macropinocytosis in neuronal cells caused ER stress and seeded the aggregation of intracellular, endogenous SOD1 (Sundaramoorthy et al., 2013).

Studies in various SOD1 transgenic ALS mouse models have provided direct evidence for WT SOD1 mediated toxicity *in vivo*. Intriguingly, an ALS-like phenotype was only observed in transgenic mice expressing the human SOD1 A4V variant, a mutation that corresponds to an aggressive ALS phenotype in humans, when these mice expressed the human WT version of SOD1 (Deng et al., 2006). This paradoxical result may be explained by heterodimerization of WT and A4V subunits, affording a “stabilized” and thus more toxic version of SOD1 A4V that would otherwise be degraded (Witan et al., 2008, 2009). A similar phenomenon was observed in double-transgenic mice expressing human SOD1 WT and G85R, where disease onset was hastened relative to single-transgenic SOD1 G85R mice (Wang et al., 2009). Recently, a transgenic mouse model was developed that over-expresses human WT SOD1 at similar levels to the established high-copy SOD1 G93A mouse model (Gurney et al., 1994; Graffmo et al., 2013). Compared to transgenic mice expressing fewer copies of the human WT SOD1 gene, the mice generated by Graffmo et al exhibit an ALS-like phenotype that includes significant weight loss, SOD1 aggregation, neurodegeneration, gliosis, and a shortened life-span of ~360 days (Graffmo et al., 2013). The authors posit that the ALS-like phenotype is not a general effect of SOD1 overexpression *per se*, but rather a direct consequence of a substoichiometric population of misfolded SOD1. Although the exact mechanism for WT SOD1 mediated toxicity is not well understood in these models, and the extent to which WT SOD1 is post-translationally modified has not been addressed, these studies clearly demonstrate a link between WT SOD1 and motor neuron degeneration characteristic of ALS.

### Evidence for misfolded, toxic WT SOD1 species in SALS

There is accumulating evidence that WT SOD1 can misfold *in vitro* and exert a toxic effect *in vivo*. That genetic mutations in SOD1 are sufficient to cause ALS raises the possibility that modified forms of WT SOD1 may cause SALS, especially because modified WT SOD1 closely mimics the toxic behavior of FALS-linked SOD1 mutants in the context of numerous assays described above. To date, there is no animal model for SALS. Therefore, investigations into the relevance of WT SOD1 in SALS are focused on biological samples from individuals with this disease.

Early immunohistochemistry (IHC) studies using pan-SOD1 antibodies detected SOD1 within Lewy body-like inclusions in spinal cord sections from individuals with SALS (Shibata et al., 1994, 1996), implicating WT SOD1 in SALS pathology. However, pan-SOD1 antibodies failed to detect SOD1 containing aggregates in every IHC study that included SALS cases (Watanabe et al., 2001). In recent years, conformation specific antibodies have been developed that discriminate between native WT SOD1 and mutant and/or misfolded SOD1. These antibodies are being employed using IHC to address whether misfolded WT SOD1 is in fact present in post-mortem SALS tissues [Figure 4, Table 1, reviewed in Bosco et al. (2011), Furukawa (2012)]. Forsberg et al generated multiple polyclonal antibodies against small peptide sequences spanning the entire SOD1 protein. Two antibodies targeted to amino acids 4–20 within β-strands 1 and 2, and amino acids 131–153 that includes both the electrostatic loop and the SOD1 dimer interface [i.e., the same sequence used to develop the SEDI antibody Rakhit et al. (2007)] were shown to detect SOD1-containing aggregates within the motor neurons of all 29 sporadic cases examined and punctate staining in only 2 out of 19 non-neurological controls (Forsberg et al., 2010). The C4F6 conformation specific antibody introduced earlier was shown to react with misfolded WT SOD1 in a subset (4 out of 9) of SALS spinal cord sections (Bosco et al., 2010), but produced a diffuse staining pattern as opposed to a punctate pattern that would be consistent with insoluble aggregates (Bosco et al., 2010; Brotherton et al., 2012). The DSE2 antibody, which recognizes a “Disease-Specific Epitope” located within the electrostatic loop that is only exposed when SOD1 is misfolded (Vande Velde et al., 2008), was shown to detect misfolded SOD1 within SALS cases that also exhibit TDP-43 pathology (Pokrishevsky et al., 2012). Although these conformation specific antibodies appear to recognize different types of WT SOD1 species (i.e., soluble misfolded vs. aggregated), these reports collectively implicate SOD1 in SALS.

However, not all SOD1 conformation specific antibodies have detected misfolded or aggregated SOD1 in SALS cases. The USOD antibody, which was generated against residues 42–48 within “Unfolded SOD1” failed to detect misfolded SOD1 in SALS cases as did the SEDI antibody, whereas both antibodies detected aggregated SOD1 in FALS (Liu et al., 2009; Kerman et al., 2010). Furthermore, some antibody studies have produced conflicting results. While Bosco et al did not detect C4F6 reactivity in 17 control spinal cord sections, Brotherton et al., reported C4F6 reactivity in SALS tissues as well as tissues from controls (Brotherton et al., 2012). The source of these discrepancies is not clear, and may stem from technical differences in the experimental procedures, inherent variations in the nature of these antibodies (i.e., different epitopes) or inconsistancies amongst the SALS cases utilized across studies.

Biochemistry-based methods offer an alternative approach to IHC for the investigation of misfolded WT SOD1 in SALS. One study reported that SOD1 present in FALS and SALS spinal cord extracts is more susceptible to forming a 32 kDa cross-linked species upon treatment with a biotinylation reagent, suggestive of a misfolded SOD1 molecule that is common to FALS and SALS (Gruzman et al., 2007). This 32 kDa species, however, was later identified as carbonic anhydrase and not misfolded SOD1 (Liu et al., 2010). Subsequently, SOD1 immunopurified from SALS spinal cord tissues was shown to inhibit FAT in squid axoplasm to the same extent as recombinant forms of both oxidized WT and FALS-linked mutant SOD1 (Bosco et al., 2010). Although misfolded SOD1 has been detected in SALS tissues with different antibodies by IHC, this study demonstrated that SOD1 derived from SALS tissues could in fact exert a “toxic” effect in an ALS-relevant assay (Bosco et al., 2010; Yates, 2010). Importantly, this effect was blocked by C4F6 (Bosco et al., 2010), indicating that C4F6 reports on the toxic region within SOD1 and therefore may be useful for designing immunotherapeutic strategies for humans with ALS.

Haidet-Phillips et al. further demonstrated the toxic nature of SOD1 in the context of SALS, as astrocytes derived from human SALS spinal cords exert a toxic effect onto motor neurons only when they expressed near-endogenous levels of SOD1 (Haidet-Phillips et al., 2011). This is consistent with the observation of misfolded SOD1 in glia (Forsberg et al., 2011). Moreover, an ELISA designed for the detection of auto-SOD1 antibodies in human sera demonstrated that these antibodies can influence the SALS disease course. Elevated auto-SOD1 antibodies reactive for the misfolded oxidized form of WT SOD1 conferred a survival benefit within the SALS cohort examined, whereas those cases with elevated auto-SOD1 antibodies against the native WT SOD1 exhibited shorter survival (van Blitterswijk et al., 2011). These data are consistent with a toxic effect of misfolded SOD1 in human SALS, but also indicate a disadvantage to lowering endogenous levels of normal WT SOD1 in SALS. Therefore, it may be necessary to avoid immunotherapeutic and anti-sense oligonucleotide strategies that reduce levels of the normal, native WT SOD1 protein.

The actual molecular nature of “toxic” WT SOD1 species within the aforementioned human SALS studies was not addressed, and therefore it is not clear whether WT SOD1 in SALS exhibits aberrant post-translational modifications. It is intriguing that the H_2_O_2_ product of the dismutation reaction that is catalyzed by SOD1 (Figure 2) can also induce the conversion of this otherwise normal protein into a misfolded, toxic species (Ezzi et al., 2007; Bosco et al., 2010). A report by Guareschi et al., may shed some light onto the nature of the SOD1 modifications *in vivo*, as elevated levels of carbonylated SOD1 species were detected in lymphoblast cell lines from a subset of SALS patients with bulbar onset (Guareschi et al., 2012). An over-oxidized form of WT SOD1 that could explain both SOD1 misfolding and toxicity in SALS is reasonable, considering that oxidative stress is a pathological hallmark of SALS (D'Amico et al., 2013).

While there is compelling data supporting that misfolded and/or aggregated WT SOD1 is *associated* with SALS, it is still unclear whether WT SOD1 can *cause* SALS. The existing data cannot exclude the possibility that misfolded SOD1 is simply a downstream effect of disease. In fact, oxidized SOD1 has been detected in the brains of individuals with Alzheimer's and Parkinson's disease (Choi et al., 2005). Moreover, the conformation specific antibodies that detect misfolded and aggregated SOD1 in SALS also detect misfolded SOD1 in SOD1-negative FALS cases as well as in the context of other neurodegenerative disorders (Forsberg et al., 2010, 2011; Brotherton et al., 2012; Pokrishevsky et al., 2012). Therefore, misfolded SOD1 may represent a general consequence of aging and disease. Nonetheless, one would still expect the presence of misfolded SOD1, whether it be the mutant or WT form, to exacerbate disease based on all of the data demonstrating the toxic effects associated with these proteins. Several therapies are under development, with some in clinical trials, that target SOD1 (Glicksman, 2011). It will be important to determine whether any SOD1-based therapies can confer a therapeutic benefit to those individuals with SALS as well as FALS. Such an outcome would provide unequivocal evidence that SOD1 is indeed a pro-active factor is SALS pathogenesis.

## Future outlook

There is an indisputable role for mutant-SOD1 in FALS; however, whether there is an analogous role for WT SOD1 in the context of SALS is unclear and controversial. The evidence that misfolded WT SOD1 is present in human post-mortem SALS samples, together with two decades worth of evidence that misfolded SOD1 can exert a toxic effect onto cells, supports the hypothesis that WT SOD1 is a causal factor in SALS. However, the presence of misfolded WT SOD1 may simply represent a downstream, non-specific consequence of aging and disease. Because SALS accounts for 90% of ALS cases, and the field is lacking an effective therapy for this devastating disease, a role for misfolded WT SOD1 in the pathogenesis of SALS should be considered. Such a role may include an upstream trigger of the disease, and/or a factor that promotes disease progression. As therapies become available to treat SOD1 in the context of FALS, it will be important to assess whether these therapies can also be applied to all or at least a subset of SALS.

### Conflict of interest statement

The authors declare that the research was conducted in the absence of any commercial or financial relationships that could be construed as a potential conflict of interest.
